# Suptypes of JIA have different susceptibility for developing pain amplification syndrome

**DOI:** 10.1186/1546-0096-9-S1-P222

**Published:** 2011-09-14

**Authors:** N Draheim, J Reckzeh, L Croce, R Häfner, E Schnöbel-Müller, JP Haas

**Affiliations:** 1 Deutsches Zentrum für Kinder- und Jugendrheumatologie, Garmisch-Partenkirchen, Germany

## Background

Primary pain amplification syndrome (PAS [Juvenile fibromyalgia]) is an important differential diagnosis in patients suspected to have JIA. Secondary PAS may occur within the course of JIA after remission.

## Aim

Characterizing the occurrence of JIA in a group of patients suffering from PAS.

## Methods

Retrospective analysis in 319 patients with PAS admitted to our unit for chronic pain in 2010. Patients` medical histories have been worked up concerning chronic inflammatory diseases. The observed frequencies of JIA subtypes have been compared to frequencies recorded by the national German registry for rheumatic diseases (Deutsches Rheumaforschungszentrum DRFZ 2008).

## Results

The medical history reported JIA in 61 patients (19%) and chronic multifocal osteomyelitis (CRMO) in 9 (3%) of the patients. (Table 1).

**Table 1:** 

	Number observed	% observed	Number expected	% expected
Oligo-JIA	20	33	32	53

Poly-JIA	23	38	9	14

Psoriasis-JIA	11	18	5	8

Entesitis associated-JIA	6	10	7	11

Systemic JIA	1	1,5	4	6

More than one third of the histories were not conclusive for the diagnosis of JIA according to the ILAR criteria, mainly because arthritis had not been assured to be present for more than 6 weeks.

## Conclusion

Polyarticular and Psoriasis JIA seem to have an increased risk to develop secondary PAS. Primary PAS has to be suspected in a number of patients diagnosed as PAS secondary to JIA as arthritis never had been proven conclusively. There are a remarkable number of CRMO patients within the group of secondary PAS patients.

